# Piloting creative engagement strategies to explore themes of parenthood with fathers

**DOI:** 10.3389/frcha.2023.1204865

**Published:** 2024-01-11

**Authors:** Iryna Culpin, Catherine Lamont-Robinson, Mark Billington, Matthew James, James Prewett, Gareth Ward, Mireia Bes Garcia, Giovanni Biglino

**Affiliations:** ^1^ Centre for Academic Mental Health, Population Health Sciences, Bristol Medical School, University of Bristol, Bristol, United Kingdom; ^2^ Department of Child and Family Health, Florence Nightingale Faculty of Nursing, Midwifery and Palliative Care, King’s College London, London, United Kingdom; ^3^ Centre for Academic Primary Care, Bristol Medical School, University of Bristol, Bristol, United Kingdom; ^4^ Independent Researcher, Bristol, United Kingdom; ^5^ Business and Civic Engagement Department, University of Bristol, Bristol, United Kingdom; ^6^ Translational Health Sciences, Bristol Medical School, University of Bristol, Bristol, United Kingdom; ^7^ National Heart and Lung Institute, Imperial College London, London, United Kingdom

**Keywords:** creative healthcare, masculinity, fatherhood, parenthood, public engagement, participatory arts-based research, ALSPAC

## Abstract

**Introduction:**

The role of the arts in health is increasingly recognised, with participatory arts-based approaches facilitating public engagement. However, little is known about men's involvement in art-based participatory research. We aimed to investigate how men who are fathers may be engaged creatively to explore experiential aspects of fathering and parenthood.

**Methods:**

Fathers collaborated with an artist, sharing individual perspectives around fatherhood by telephone and email, leading up to creative representations of fatherhood. Initial conversations were prompted by images from a 2020 exhibition catalogue entitled “Masculinities” (Barbican Centre, London) inviting participants' responses to the photographic curation. The catalogue served as an artistic reference to gauge a sense of participants' creative predispositions, as well as a foundation to facilitate spontaneous dialogue about personal meanings of fatherhood. Fathers' experiences of contemporary arts varied greatly; yet all fathers confidently shared responses ranging from photographers' representation of masculinity and fatherhood and perceptions of what was excluded or privileged within this very specific curation. These discussions further led to conversations around representations of fatherhood and highlighted particular areas of interest in terms of fathers' involvement in research and public engagement. The artist provided reflections to each participant by email with links to arts resources building on the initial conversations. Two further shorter sessions followed as fathers' key messages emerged, and the final forms of their own creative expressions crystallised.

**Results:**

The final pieces included a musical composition around sharing vulnerability as a new father, a word cloud to represent gendered language of parenthood, an animated graphic image representing the bond between father and child, a combination of short poetic stanzas highlighting assumptions around fatherhood, an experiential photographic record of a father and a son in the early years, and a cartoon strip around emotional intelligence in parenting.

**Discussion:**

Arts-based participatory engagement enabled to capture deep-rooted experiences of being a father in modern society, illuminating common cultural and intergenerational perspectives, while also tapping into unique individual experiences. The richness and diversity of these unique responses suggest that arts-based methodology can facilitate public engagement with men and lead to deep reflections on complex and socially constructed phenomena such as fathering and parenthood.

## Introduction

The role of the arts in health is increasingly recognised and evidenced, with participatory and creative approaches offering new ways to generate and disseminate knowledge in health and social research (1, 2). Arts-based methods have the potential to elucidate subjective health and social dimensions of human experience, in ways that complement and augment existing qualitative methodologies (3, 4). It has been argued that arts-based approaches expand on qualitative research methodologies through additional representational possibilities emerging in the creative process and sensory engagement required from participants and audiences (1, 5). Arts-based research also helps to highlight those aspects of lived experiences that are often overlooked in interviews and focus groups due to the increased participant-led nature of such methods in producing arts-based work (6, 7).

One area in which it is valuable to explore the potential of creative approaches to complement other research methods is the examination of the role that fathers play in child development, including those families where mothers experience postnatal depression. This is the subject of a research programme that combines sociological, epidemiological and developmental methods (led by IC, funded by the Wellcome Trust) to disentangle complex processes that underly transmission of mental health risks in families. The sociological aspects of the study focused on elucidating the nature of fathering and social processes that shape father involvement, while epidemiological and psychological approaches examined the effects of maternal postnatal depression on the child and the role of fathers' parenting and involvement in this context using longitudinal and behavioural-observational data on parenting and father-child interactions. The intergenerational transmission of mental health risks from parents to children is complex and multi-factorial, calling for integration of interdisciplinary approaches. In particular, the role of fathering in families affected by depression remains largely unexplored from sociological, epidemiological and developmental perspectives. Combining inter-disciplinary methodological approaches enables the analysis of processes underpinning family functioning in the context of perinatal mental health, particularly the nature and dimensions of fathering and its impact on child development. The in-depth sociological examination of the impact of maternal postnatal depression on fathers' experiences of parenting and involvement aimed to generate insights into how fathering is “co-created” and negotiated in the context of family dynamics affected by depression. This includes the development of different forms of fathering in terms of emotional and practical involvement, as well as unique insights into the impact of maternal depression on parental relationship, fathers’ own mental health and the nature and quality of their interactions with children. Substantial epidemiological and sociological literature has now emphasised the important role that fathers' involvement and parenting play in child development (8–10), while paternal mental health has increasingly been linked to various emotional, behavioural and cognitive dimensions of child development in its own right (11), as well as in the context of maternal postnatal depression (12).

Participatory arts-based research can be a powerful way to unsettle limiting hierarchies and challenge representations (13, 14), but also to share experiences, build connections, and promote social change. Ball et al. (15) in their extensive review of arts-based approaches for public engagement with research, emphasize how the design and delivery of community interventions requires acute sensitivity to the cultural, political and socio-demographic context. Multiple forms of knowing, such as sensory, kinaesthetic and imaginary (16) have long been valued within practice-based research, as articulated by Sullivan: “*Such making is not just doing, but it is a complex, informed, physical, theoretical and intellectual activity where private and public worlds meet. Art practice is the outcome of intertwined objective, subjective, rational and intuitive processes*”. (17, pp. 78). By engaging fathers in participatory arts-based research, we were hoping to elicit visceral, embodied and imaginative interpretations of fatherhood and parenthood; experiences which are often obscured by societal expectations and not fully captured by traditional sociological forms of enquiry.

We thus aimed to explore how fathers may be engaged creatively to explore complex and experiential aspects of fathering and parenthood beyond verbal accounts. Importantly, arts-based methods may be most suitable for engaging those fathers who are less confident in verbal expression to explore the nuances of parenting. Fathers' voices are lacking in research, and we hoped to empower them to share their stories through creative channels of communication and engagement, capturing their individual and collective experiences of parenting in a wider context of societal perceptions of masculinity and gender. These accounts may have profound implications for our understanding of nuanced facets of father-child relationship that may be channelled into development and delivery of prevention and intervention programmes that strengthen father-child relationship and improve child development. The larger study provided a *context* and *framework* for this explorative work, primarily focused on the arts-based engagement methodology, as well as a mechanism for participant recruitment.

Despite the growing popularity of and evidence on arts-based methods in sociological, psychological and health research, very little is known about men's involvement in arts-based participatory public engagement. Here we investigate how men who are fathers may be engaged creatively to explore experiential aspects of fathering and parenthood, elaborating on their meaning through engagement with arts and individual interviews. Arts-based research is perfectly positioned to raise awareness and provide a platform for expression and meaning making for those individuals who have been traditionally less involved in research, including fathers (18). It enables to explore multiple dimensions of human condition, including fathering and parenthood, from the emotional, social, cultural and physical perspectives, reflecting myriad of ways of engaging in the world (19). By using an arts-based approach we hoped to extend methodological techniques to engage men in research while addressing social relationships, norms and expectations that shape experiences of fatherhood, exploring aspects of fathering that thus far remained inaccessible.

## Methods

### Participants

The sample comprised participants from the Avon Longitudinal Study of Parents and Children (ALSPAC), the Children-Generation2 (ALSPAC-G2) cohort, which was set up to provide a unique multigenerational cohort, building on the existing ALSPAC resource of originally recruited women and their partners (Generation 0; ALSPAC-G0) and their offspring (ALSPAC-G1) followed up for 26 years. Recruitment of the next generation ALSPAC-G2—the grandchildren of ALSPAC-G0 and children of ALSPAC-G1—began on 6th June 2012. Up to 30th June 2018, 810 ALSPAC-G2 participants from 548 families had been recruited. Over 70% of those invited to early- and late-pregnancy, second week of life, 6-, 12- and 24-month assessments attended, with attendance >60% for subsequent visits up to 7 years. Further details on the cohort profile, representativeness and phases of recruitment, including ALSPAC-G2, are described in four cohort-profile papers (20–23). ALSPAC study website www.bristol.ac.uk/alspac/ contains details of all the data that is available through a fully searchable data dictionary and variable search tool (http://www.bris.ac.uk/alspac/researchers/our-data/). Informed consent for the use of data collected via questionnaires and clinics was obtained from participants following the recommendations of the ALSPAC Ethics and Law Committee at the time.

On 22nd July 2019, through additional funding from Wellcome Trust, a separate research clinic for fathers was set-up (Focus on Fathers) inviting fathers directly to attend a range of assessments when their G2 child was six months old. In order to diversify the sample, fathers were also recruited through the community using a variety of mechanisms, including media advertising and study advertisements. Participants who attended ALSPAC-G2 research clinic (*n* = 3) and those fathers recruited from the community (*n* = 3) into the larger study were contacted at a later stage to participate in the arts-based participatory study. There were no specific inclusion/exclusion criteria to recruit fathers into the creative engagement study other than previous participation in the larger study (Focus on Fathers). Fathers were approached randomly using their fully anonymised Identification Number (ID).

### Ethical standards

Informed consent for the use of data collected via questionnaires and clinics was obtained from participants following the recommendations of the ALSPAC Ethics and Law Committee at the time. Further ethical approval was sought to conduct arts-based participatory study from the ALSPAC Ethics and Law Committee (approved 12th October 2020). Written informed consent was obtained from the participants for the publication of any potentially identifiable images or data included in this article.

### Planning and running creative sessions

Six fathers (*n* = 6, age range: 29–65) were invited and agreed to participate. All fathers were White British and in full-time employment. The majority of fathers reported university level qualifications, except one father who reported A-Level qualifications. All fathers, except one who was separated, were married to the mother of their child. In light of their prior engagement in this research, an ethical framework was already in place, however additional project considerations included the public dissemination of participants' final works (24) and the opportunities and challenges of digital platforms (25). Participants were invited to join individual artist-led sessions, conducted remotely, to elaborate creatively their recent experience of fatherhood. Participants were reassured that there was no requirement for previous arts experience, aiming to creative a safe and inclusive space and moderating participants’ expectations and potential anxieties. The role of the artist facilitator in creatively supporting individual, creative explorations of fatherhood and parenthood was highlighted as central to the ethos of the project. The sessions were conducted individually, rather than in a group setting (which was also considered), to allow for nuanced individual responses to emerge. As Archibald & Blines (26) suggest: “Arts-based health research offers unique opportunities to integrate evidence of patients’ lived experience with other forms of research evidence to improve understanding and knowledge translation, but transparent descriptions of this praxis are generally lacking”. Throughout our mapping of the creative sessions, we were attentive to providing an inclusive ethos designed to support diverse, unique artistic perspectives. We hoped that trust forged in earlier research involvement indicated authentic appreciation of participants' realities and knowledge systems (27) and aimed to provide opportunities for further reflection which may not surface in interview-based methods (1).

Sessions were run in November/December 2020—January/February 2021 and Covid-19 restrictions throughout the period of the public engagement project excluded face-to-face contact. We therefore drew on remote technologies and devised a framework for virtual creative 1:1 sessions led by the artist (CLR). As a prompt for conversations and an icebreaker for the creative sessions, each participant was sent a copy of “Masculinities: Liberation through photography”, the catalogue of the eponymous exhibition at the Barbican Art Gallery (London, 13 July-23 August 2020), a major group show exploring how masculinity is experienced and constructed as expressed and documented through over 300 works by artists, photographers and filmmakers such as Richard Avedon, Peter Hujar, Isaac Julien, Robert Mapplethorpe and Catherine Opie (https://www.barbican.org.uk/our-story/press-room/masculinities-liberation-through-photography). The Barbican website contains a detailed description of the exhibition, including the pre- and installation images, which provide visual examples of exhibited work. The artist considered the catalogue as a shared creative launching point to facilitate the dialogue. “Fatherhood” and “masculinity” are distinct, yet related concepts that have dominated scientific and public discourse that examines their possible connections to child outcomes and to outcomes for fathers themselves (28). Our choice of catalogue was prompted by the conceptual intersection of these constructs (e.g., Fatherhood-Masculinity Model (28); to initiate and facilitate the dialogue. However, we did not intend to interrogate the distinctions and similarities of these concepts as part of the engagement process, neither did we aim to frame our engagement with fathers in any particular conceptual framework. The choice of the catalogue as a creative prompt was also driven by the practical necessity to adapt face-to-face and in-person engagement methodologies in light of the Covid-19 public health and lockdown restrictions.

Individual informal phone conversations between the artist and fathers were jointly scheduled and, increasingly, participant-led. The catalogue and imagery contained in it served as a launching point to discuss arts representation in general, effectively representing a shared visual and conversational reference. The artist requested each participant's permission to informally document dialogues, then offer reflections plus artistic resources by email following each phone-call.

Results are presented as reflections on each participant's narrative, including quotes and own writing as well as a creative output for each.

## Results

The six individual narratives and corresponding imagery emerged from the creative process are presented individually. Each has been given a title (by the research team) to encapsulate the narrative.

### “Wearing your heart on your sleave” (Gareth)

Gareth was intrigued by the span of the historical, stylistic and content within the catalogue archive. Images he selected to discuss included close-up explorations of facial expressions, interpersonal compositions across generations, eras and socio-cultural perspectives.

This led to reflections around being a positive role-model for his children—challenging gendered and stereotypical expectations both on the domestic and work front (“*it does frustrate me that in a time of equality fathers’ rights seem unequal*”) as well as assumptions around “*natural parenting*”.

Gareth followed up emerging personal themes between phone calls through digital searches [“*images can appear perfect with proud, confident dads*, (…) *underneath there can be anxieties… I feel most males are likely to hide these despite it being normal*”] including sharing: “Melancholy” by Albert Gyorgy, a large-scale, seated, bronze figure with head hung low and a gaping hole in the torso—shared as a symbol of emotional vulnerability; a poignant animation represented “*fears for children's futures in this ever -changing world*”; Our Smartphone Apocalypse by Steve Cutts, which mirrored Gareth's witnessing of interpersonal disconnection between parents and children through habitual mobile-phone usage; dramatic compositions from The Cinematic Orchestra, which were exchanged to illustrate the power of music to hold contrasting emotions in balance.

**Figure 1:**
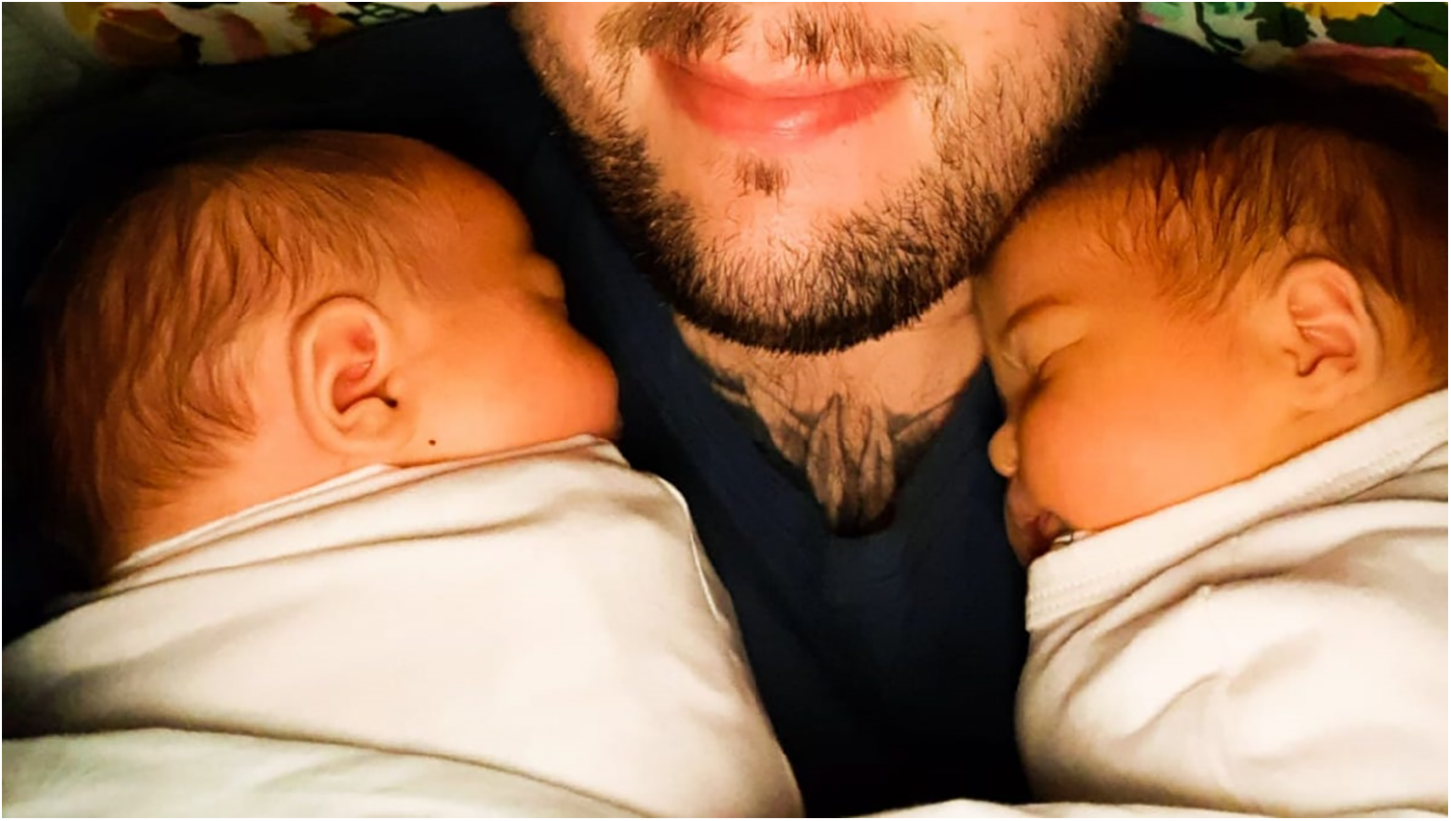
Photographic creative output from one of the participants (Gareth).

Gareth chose to share an image as his creative piece (Figure 1), adding: “Fatherhood is an emotional journey and that it is okay. It doesn't make you weak. I've cried more in the first three years of my children's life than in the last 20 years”. He hoped that this project would also invite mothers to “have more awareness—as I have hidden it away from my wife”. He further suggested “having children brings an enhanced emotional intelligence with males, which I don't think they necessarily know how to discuss or channel… To have the best thing in the world happen to you, having this array of such strong feelings includes love, anxiety, and sadness is hard to control…” and hoped that sharing this “rollercoaster of emotion …sadness and embracing joy and dads allowing themselves to cry through both… would be helpful to other fathers”.

Digital arts and multi-media provided the creative resources to extend Gareth's dialogue and reflections across this project as opposed to studio-based workshops. Gareth's choice to capture an intimate image which viscerally represented his close bonds as a father perhaps points to a more nuanced way forward regarding social media—organically incorporating technologies whilst celebrating the relational qualities of inter-personal attention.

### “Different points of view” (Matthew J)

Conversations around seemingly “fixed” representations of masculinity in the Barbican catalogue inspired discussion around “*the impact of location and upbringing*” in identity construction and the lure of “group/gang” mentality even in the early years “*drawing boys into stereotypical behaviour patterns which limits their life experiences and abilities to connect and contribute*”. Matthew J however suggested that beneath “*this veneer* of *washed-out individuality*”, identity is still fluid. He advocated “*stepping back and cognitively processing information before emotionally responding*” to foster agency when males are feeling “*voiceless*”.

Matthew J related this strategy to his current roller-coaster of experiences as a new parent and highlighted the importance of “*supporting fathers to find their own ways relating to their children—which may be complementary to those of their partners but equally valuable in models for future generations*”*.*

**Figure 2:**
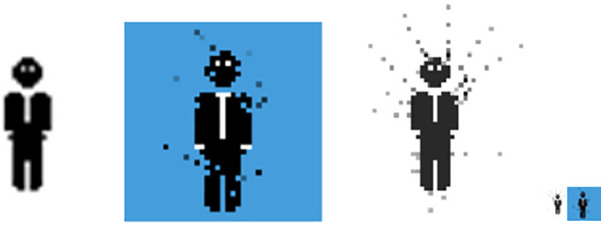
Small animation reflecting on pixelation and idea of rebuilding, as developed by one of the participants (Matthew J).

As to his creative output, he created a digital animation (Figure 2) and added: “*I am very interested in pixelation as an analogy for life in that we only ever see a small part of the big picture and cannot really ever understand how it all fits together because we can’t get far enough away from it. Those ideas feel very relevant to me now as well as the concept of being broken up and rebuilt which, for me, is what becoming a dad has been like. I’ve also included a small animation. Its small in length and size! The size is an experiment. Playing with at what point is it not readable—but I also want to convey the overwhelming scale of parenting by shrinking the individual. I wanted to represent the breaking up and rebuilding in motion as well as in still-images*”*.*

### “Permission to express emotions” (James)

James discussed an individual and group portrait from the Barbican catalogue in depth highlighting a Welsh coal-miner's eyes shining with the warmth of humanity amidst ground-in coal dust and the dissonance of banal, shared moments whilst in the full battle accessories during a Taliban group's down-time. The following conversation surfaced a desire to strip-back gendered, cultural, social and global differences, including separations between human/non-human and respect what unites us all. James later reflected on the negative impact of assumptions, prejudice, historically and culturally-rooted perspectives within parenting and suggested crafting some thought-provoking phrases or short poems to generate a wider debate. Reflecting back on the process of writing, James noted that his words seemed to have morphed into rap verses …he found himself drawing directly on his own parenting “*some parts are more angry than I expected* …*but I just thought I would write it all down*—*then edit bits out at a later date*”. The following verses are the first two and final paragraphs:

### Second chance

I think about my parents and hate some of their choices

Growing up to a chorus of so many raised voices

Never had an opinion or ever complained

Still think it affects me and keeps me restrained

When I sit alone, lost in reflections

Were these reasons or just deflections?

Our family thrived on restraint of emotion,

Hell no would we be honest and cause a commotion

When I'm with my family it feels I've woke from a dream,

The thought of a family this happy at one point was obscene

I'd never want my daughter estranged for some time

She completes our family like rhythm pairs rhyme

In conversations, James had focused on what he and his partner hoped for their family, therefore stanzas about his father such as “*Did you ever show affection without us having to ask*?” provided a stark and hard-won emotional literacy illuminating the potential to reconfigure negative experiences of fatherhood.

### “Finding the right words” (Matt)

**Figure 3:**
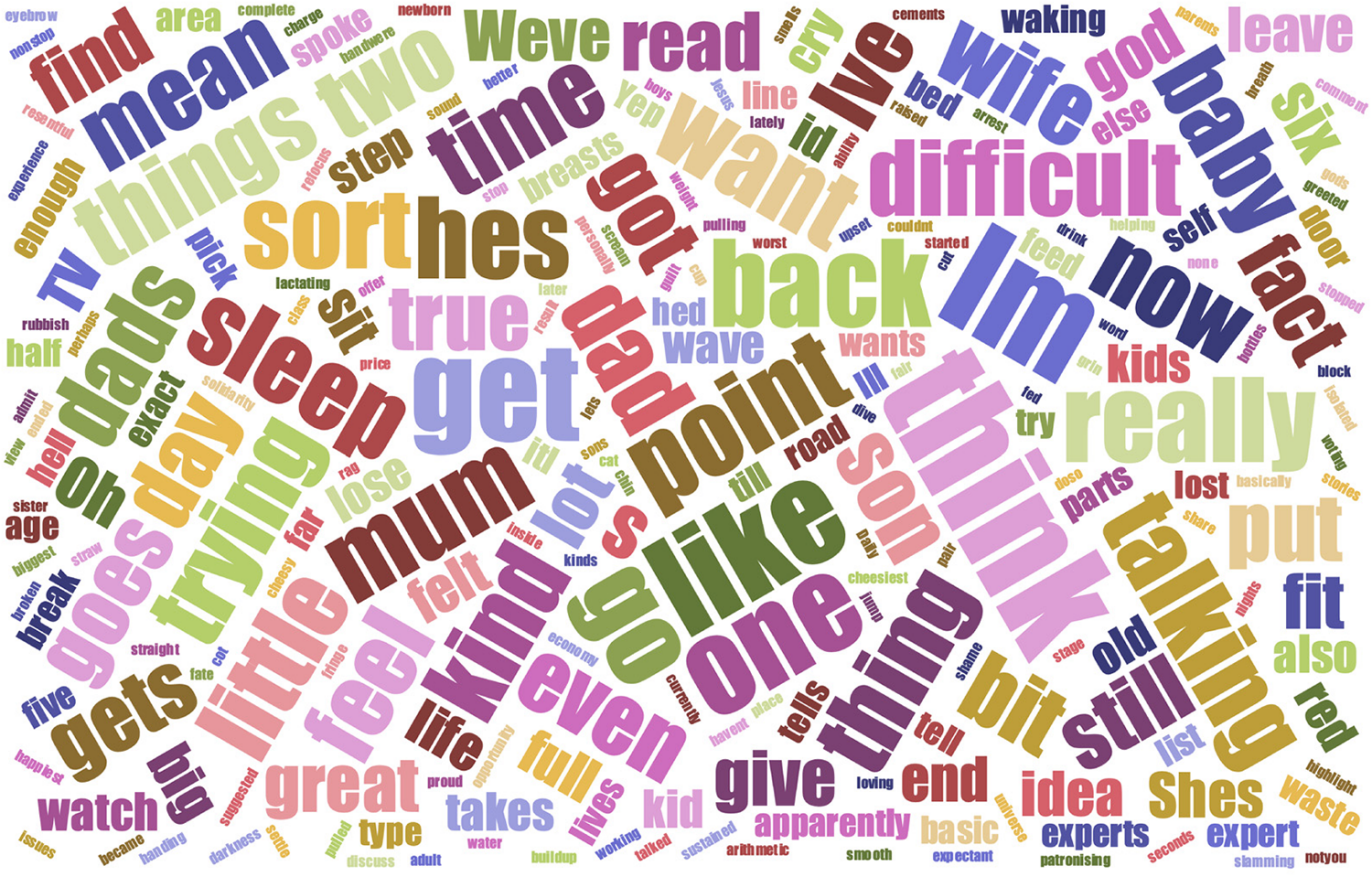
Word cloud resulting from analysis of “First time dad” podcast episode, to encapsulate what becoming a dad means, as developed by one of the participants (Matt).

The Masculinities catalogue triggered Matt to reflect on the evolution of gender assumptions and expectations across the decades with a lack of “three-dimensional male role-models” in mind. He referenced several contemporary images which highlighted the ongoing legacy of socio-culturally prescribed behaviours such as portraits of bullfighters and fraternity rituals. Voicing the need to “*break down divisions, broaden the notion of masculinity and present healthy versions for young males in early years and adolescence*”*.* Matt felt strongly that the visual arts could provide a powerful medium in shifting public perceptions and raising new awareness—he recalled visiting an exhibition of “*totally convincing yet completely incorrect maps*” and talked about creating images which would subvert false realities by mapping out in-the-moment, lived experience of fathers. Musing that perhaps the most pervasive influences were embedded in everyday language “*which children absorb from birth*”, he considered developing two, digital word-clouds based on an analysis of parents' gendered speech around bringing up their children. Having underestimated the time and availability of relevant digital sources, Matt subsequently decided to focus on the “First Time Dads” podcast series and selected the episode “Dad guilt, Dad rage, Dad frustration, Dad envy and all the other Bad Dad feelings” for his digital analysis (https://podcasts.apple.com/gb/podcast/first-time-dads/id1297363179). As a result, he created a word-cloud (Figure 3) hoping that it might inspire debate around the impact of gendered communications and “*what it means becoming a dad*”.

### “Let them lead the dance” (Matthew C)

Matthew C noted the powerful visual iconography of the Barbican curation, citing “*extreme*” examples of masculinity such as body-building, and the minimal representation of family life which he felt “*undermined ordinary fatherhood—everyday Joe doing their best*…”. He went on to suggest that representations of parenthood in the social media often portrayed “*idyllic*” imagery—“*an inappropriate yard stick*” leading to unhelpful comparisons. He suggested: “*…most of the time the vast majority fall somewhere in the distinctively average band of people that will make as many mistakes along the way as their children and ultimately still prove to be successful parents*”.

Matthew C shared delight in “*closely observing your child's naturally emerging interests and personality*”*.* Conversation around the stimulus of different environments brought to mind his own interest in natural structures as a young child. This led to sharing images of child-led engagements from participatory arts projects. Documentation of children's creative responses to the natural environment rekindled a desire to design “*an activity board*” for Matthew's son and triggered happy memories of handling materials and tools alongside his carpenter grandfather. “ *J always seems to be happiest when he is with us and doing the same thing…he clearly learns so much from imitating … I look forward to continuing to spend time encouraging J to try new things as his interests develop*”.

**Figure 4:**
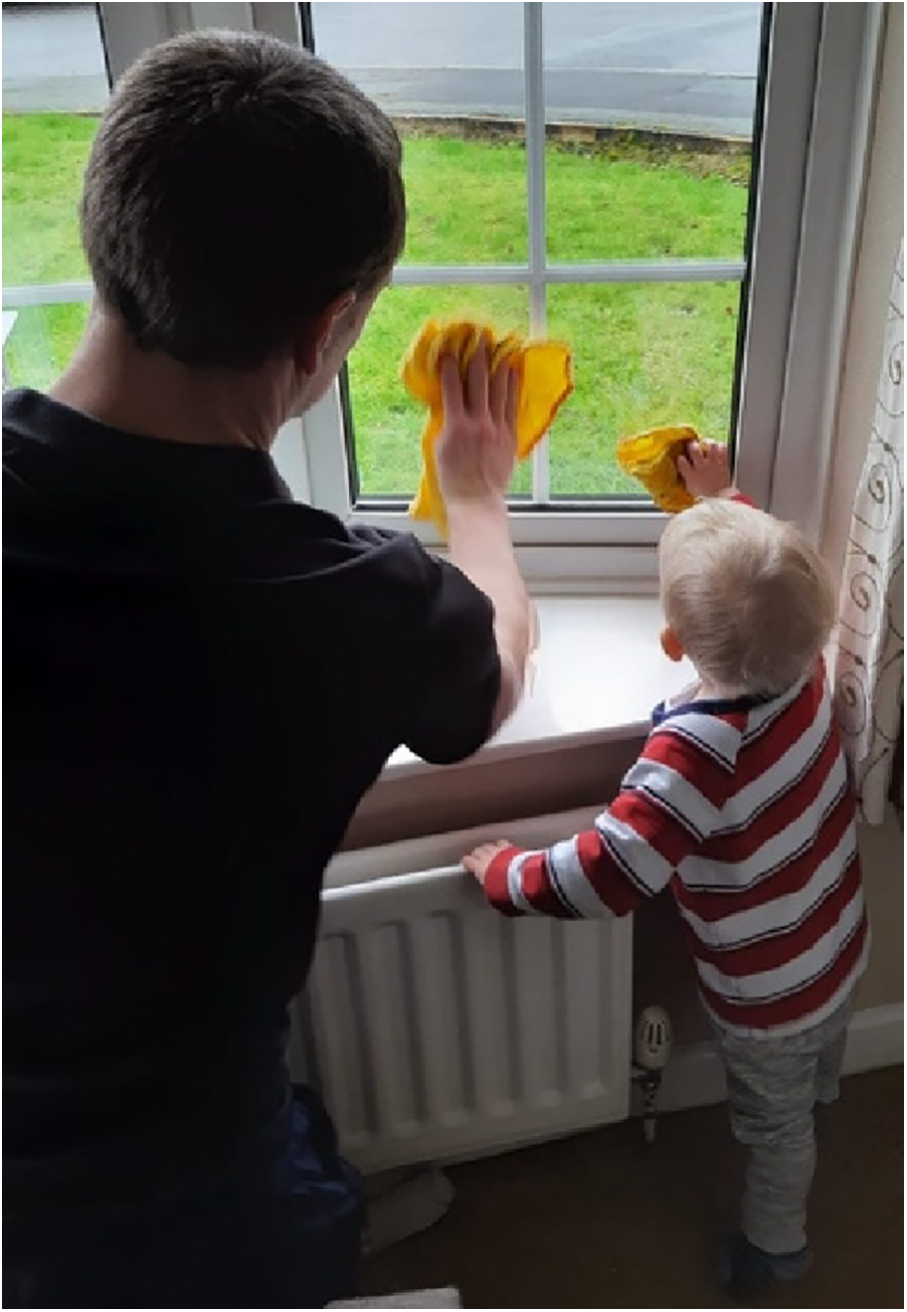
Photographic creative output from one of the participants (Matthew C).

Ultimately Matthew C's perception of his parental role was very clear—“*supporting their journey down whatever path they chose—so that they would find their place in the world and flourish in it* … *an engineered childhood could be a very unhappy one*” as reflected in his choice of creative output with a photograph encapsulating a moment of everyday life (Figure 4).

### “More than the sum of the parts” (Mark)

Mark was disappointed that a contemporary exhibition around masculinity featured such a minority of images around being a parent and felt the curatorial team lacked vision in representing “Masculinities”. He selected a range of images to discuss—a young man objectified in the process of the photographer's aesthetic process, a tender and universally relatable series around a grandfather's peaceful death, and an uncomfortable piece raising gendered power dynamics between father-photographer and daughter-model. This level of ethical scrutiny, informed by a strong sense of justice and humanity was present in how Mark valued fatherhood and his wish to challenge limiting, gender-specific assumptions and expectations.

**Figure 5:**
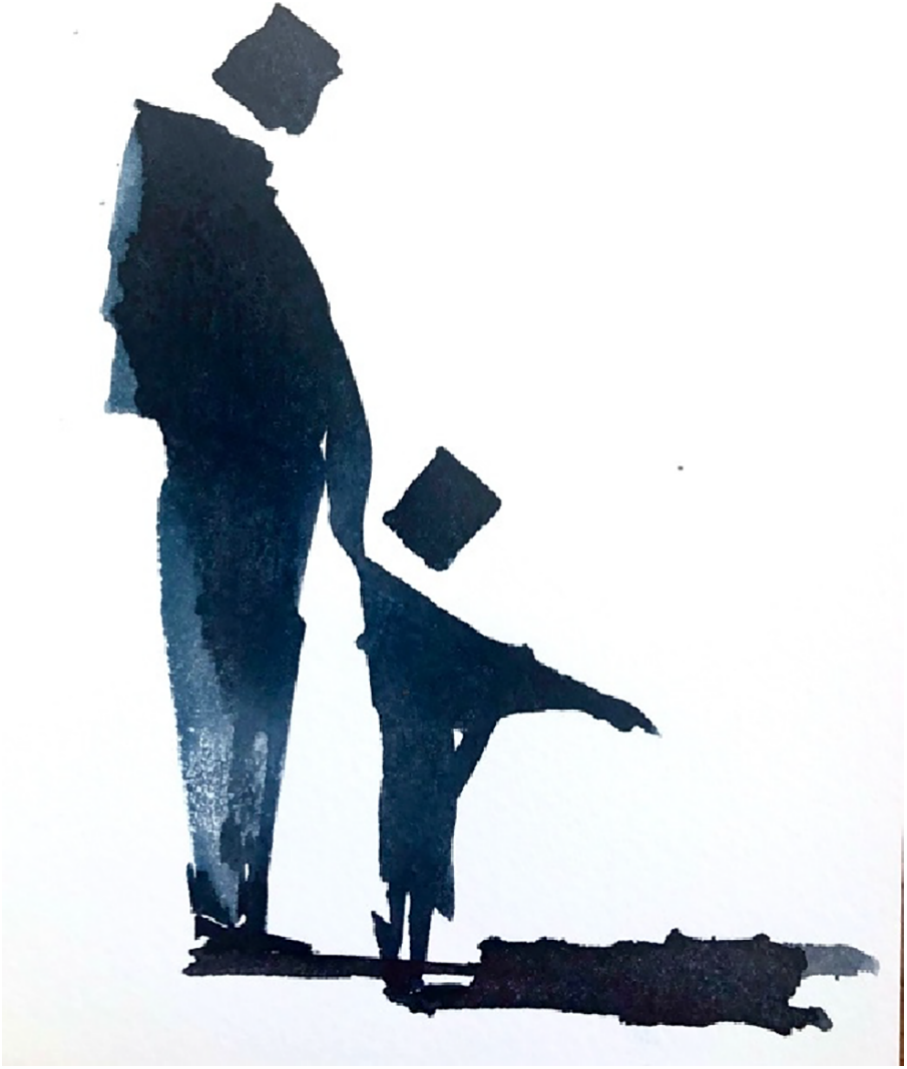
Watercolour realised by one of the participants to creatively encapsulate the concept of fatherhood (Mark).

Mark discussed the values of complementarity within parenting—providing a child with a balance of approaches and experiences and provided the painting below (Figure 5). “*This image was really just a quick doodle trying to allude to the value of fatherhood and what they can bring to the party. After viewing the catalogue of the photo exhibition about masculinity and our subsequent discussion. It seemed fatherhood didn’t seem to have been part of the curators*” *agenda or hardly represented. I just feel that fatherhood should surely be one of ultimate expressions of masculinity'.*

## Discussion

Creative engagement through imagery and reflection was conducive to rich and nuanced exploration of themes of fatherhood. When approaching such delicate and socially relevant topics, Gerber (16, p. 159) suggests that, in terms of social justice and impact, arts-based processes are: “well suited to dislodge, expose and address cultural, social and political structures in a transformative way that can affect how we perceive and respond to entrenched systemic issues”. The arts-based participatory engagement enabled us to capture deep-rooted experiences of being a father in modern society, illuminating common cultural and intergenerational perspectives, while also tapping into unique individual experiences and meanings. In line with existing literature, fathers found arts-based methods used in this study engaging and empowering in accessing deeper emotions and theme surrounding fatherhood, with the process of co-creation providing an opportunity to reflect on their experiences often placing them in the context of their own upbringing and wider of socio-cultural perspectives. The richness and diversity of these unique responses suggest that arts-based methodology can facilitate public engagement with men and lead to deep reflections on complex and socially constructed phenomena such as fathering and parenthood. An arts-based approach also has the potential to inform intervention programmes, and has implications for practitioners (e.g., community support groups for men and fathers) and policies aimed to support fathers through transition to parenthood, contextualising these experiences in wider sociocultural perspectives on gender and masculinity.

From methodological standpoint, the use of an arts-based approach complemented, enriched and expanded upon the conventional qualitative methodology (i.e., in-depth qualitative interviews) employed in the main study to explore fathers' experiences of parenthood in the context of their partners' mental health (themes that emerged as part of this inquiry will be a subject of a separate publication). The opportunity for creative expression tapped into the dimensions of these experiences beyond verbal expression, providing additional dimensions and texture to the sociological enquiry by generating nuanced data and themes. Fathers' artistic expressions (through photography, drawing, writing, animation, or even creating a word cloud) served as reflections into the lived experiences of fatherhood and parenthood. The arts-based methodology was also a way of enhancing engagement in the process of knowledge creation, as well as enhancing artist–participant communication and facilitating conversations and reflections during phone interviews, generating data that was beyond the normal scope of qualitative interviews alone (29). In line with Jones (30), we argue that arts-based methodology used in our study allowed for intersubjectivity, artistic encounter (here mediated by technology), and the collective elaboration of meaning to deepen our understanding of fatherhood and parenthood in the context of wider themes pertaining to masculinity and gender. In line with previous research, we found that creative engagement illuminated aspects and dimensions of fatherhood that may be too intricate and nuanced to capture in words (31).

The richness and breadth of themes and creative outputs that emerged as a part of this participatory engagement project (e.g., emotional vulnerability of fatherhood, identity formation as a father, emotional experiences of own childhood, socially constructed language of fatherhood, gender specific assumptions surrounding fatherhood) have also highlighted fathers' awareness of their role in their children's development and their unwavering desire to be part of this journey in practical and emotional ways. The idea of “intimate fathering” that encompasses but goes beyond an emotional connection, prioritising the quality of parent-child relationship, has been strongly advocated in contemporary sociological literature (32, 33). Father involvement and quality of father-child relationship (34) have important implications for numerous aspects of child socio-emotional, behavioural and cognitive development (35, 36). Paternal mental health may disrupt the emotional quality of father-child relationship (11), which, in turn, has deleterious consequences for healthy child development (12). Thus, it is important to support fathers through family-based prevention and intervention programmes that address fathers' mental health and strengthen family cohesion, including father-child relationship.

From a practical standpoint, the thematically relevant Barbican exhibition catalogue “*Masculinities: Liberation through photography*”, albeit focused on masculinity and not parenthood, provided an invaluable resource to broadly discuss arts representations and interpretations of these related yet distinct constructs. This group of participants were already confident in volunteering insights verbally, yet open to exploring and translating their perspectives around fatherhood through mixed/multi-media. The three scheduled phone-calls to each participant allowed the artist to engage in generative listening building a sense of creative pre-dispositions, motivations and unique life-experiences throughout the project. Email communications which ran in parallel with the phone-calls provided an opportunity for the participants to check in with the artist's interpretations of the dialogues. Devoid of a studio context, this reflexive strand also allowed the artist to re-calibrate the focus and selection of arts references to support (if appropriate) emerging creative enquiries. The open-ended, dynamic ethos of this process side-stepped limiting expectations of specific creative outputs.

As previously argued, this kind of participatory creative work also presents possible challenges (24). Alongside possible ethical issues relating to privacy and risks of identification, which are mitigated by ensuring appropriate consenting, participants' wellbeing is a key aspect as the creative exploration of feelings and experiences (including potentially unexpectedly painful ones) can represent “dangerous emotional terrain” (37). The artist as a facilitator aims to ensure that appropriate signposting and support is in place during and after the engagement activities. The wellbeing of the facilitator is also an important consideration, with the whole project team providing a space for them to reflect and appropriate support if necessary. It should also be mentioned that creative expression is open to multiple interpretations and meanings. In order to address this challenge, we have invited fathers who took part in the study to reflect on meanings and interpretations of their creative engagement and expression prior to publication. The challenge of balancing artistic and scientific components as part of arts-based enquiry has also been articulated (38). In this instance, the artist (CLR) was not familiar with previous research and fathers’ individual circumstances. Thus, they were able to establish a relationship as creative companions free of shared history, providing an opportunity and space for creative exchange and in-depths exploration of unique personal experiences and meanings. It has been argued that arts-based research produces a less tangible knowledge that can be tested for reliability and validity (39, 40). However, in our experience, engaging with creative arts-based co-enquiry opened up avenues for capturing rich and textured facets of fathers' emotional experiences of parenthood, embedded in personal experiences of being parented and wider societal and gender expectations.

It should be noted that our study took place during the Covid-19 pandemic, with emerging qualitative studies highlighting profound implications that public health restrictions exerted on fathers' experiences of becoming fathers (41), perceptions of their role (42) and parenting experiences (43). However, the effects of the Covid-19 pandemic on fathers' experiences of fatherhood and parenting were not the focus of the conversations between the artist and the fathers, thus, these themes and reflections did not emerge in our data. The small, and potentially selective nature, of our sample size should be highlighted as a potential limitation. The breadth and richness of the participants' experiences of fatherhood and parenting may need to be explored further using arts-based research methods in a larger and more diverse sample of fathers from various socio-economic, ethnic and cultural backgrounds. Nevertheless, despite these challenges and potential limitations, we argue that creative and arts-based participatory research methods have much to offer in generating profound insights into multi-faceted and complex phenomena such as fatherhood, parenting and mental health beyond “cognitive ways of knowing”, stimulating a broader perspective while engaging fathers in research (40, 44). Furthermore, the aim of this study was to focus on exploring the appropriateness and potential of these creative approaches specifically with this demographic and indeed the richness and insightfulness of the creative outputs is a clear demonstration of the value that such approaches can offer in this context.

## Data Availability

ALSPAC data are available through a system of managed open access. The study website contains details of all the data that is available through a fully searchable data dictionary and variable search tool data dictionary. The application steps for ALSPAC data access are highlighted below.
1. Please read the ALSPAC access policy, which describes the process of accessing the data in detail, and outlines the costs associated with doing so. 2. You may also find it useful to browse the fully searchable research proposals database, which lists all research projects that have been approved since April 2011. 3. Please submit your research proposal for consideration by the ALSPAC Executive Committee. You will receive a response within 10 working days to advise you whether your proposal has been approved. If you have any questions about accessing data, please email alspac-data@bristol.ac.uk. Requests to access the datasets should be directed to alspac-data@bristol.ac.uk.
